# Cannabis Stigma and Symptom Management Considerations in Cancer Survivors: A Mixed-Methods Exploration of Patient Perspectives

**DOI:** 10.21203/rs.3.rs-7244150/v1

**Published:** 2025-10-01

**Authors:** Sera Levy, Salimah Meghani, Brooke Worster, Colleen Kilanowski, Danielle Smith, Amy A. Case, Rebecca L. Ashare

**Affiliations:** State University of New York at Buffalo; University of Pennsylvania School of Nursing; Thomas Jefferson University; State University of New York at Buffalo; Roswell Park Comprehensive Cancer Center; Roswell Park Comprehensive Cancer Center; State University of New York at Buffalo

**Keywords:** cannabis, oncology, stigma, symptom management

## Abstract

**Purpose.:**

This study aims to assess perceived stigma among CS who use or consider use of cannabis.

**Methods.:**

This study employed a convergent, parallel mixed methods design utilizing focus group and questionnaire data to assess the presence of stigma among a sample of CS (n = 23) who use (n = 10) and do not use (n = 13) cannabis to manage symptoms. CS were recruited from a multi-site observational study in the Northeast U.S. region that assesses cannabis use among oncology patients.

**Results.:**

A total of 23 CS participated in this study. In general, this sample appeared to have positive attitudes towards cannabis, as indicated by quantitative results, and most CS felt accepting or neutral about other CS using cannabis, irrespective of whether they used or not. Most CS did not indicate experiences of stigma for cannabis use, did not feel judged by their medical providers, and indicated a feeling of empowerment to do whatever was needed to feel better. However, several CS reported intentional nondisclosure to their providers. Many CS discussed the presence of opioid-related stigma, both perceived from society and internalized, which appeared to play an important role in their symptom-management decision-making.

**Conclusions.:**

Findings from this study suggest that while cannabis stigma may not be commonplace for CS, some do experience it. Further, opioid stigma appears to be widely perceived and intertwined in the decision-making processes for CS.

## Introduction

Managing cancer and treatment-related pain is important for patient well-being.^1^ Although pharmacotherapy, like opioids, has been the cornerstone of managing cancer-related pain, unfavorable side effects, and fear of addiction and opioid-related stigma may deter cancer survivors (CS) from using opioids.^2–6^ Due to opioid-related concerns, CS may look for alternatives to manage pain, including over-the-counter medications and/or cannabis. ^2,7–9^

Despite federal classification of cannabis as a Schedule I substance, 39 states have legalized cannabis for medicinal purposes, and 25 for adult recreational use.^10^ Although the prevalence is growing, an estimated 40% of CS report cannabis use.^11–13^ The rapidly changing legal landscape appears to coincide with changes in the public perception of cannabis use.^14^ However, conflicting regulatory policies regarding cannabis at the state and federal levels likely contribute to ongoing stigmatized views and non-legitimate perception of cannabis, in addition to other contributors (e.g., politics, religion, etc.).^15,16^

Stigma is a social phenomenon defined as “an attribute that is deeply discredited” and, according to several models, involves four components: labeled differences, stereotypes, separation, and power asymmetry.^17^ Labeling refers to the group being identified by a common name (e.g., “cannabis users”), stereotypes are the attached negative attributes (e.g., lazy, druggies, etc.), separation refers to an “us” versus “them” ideation (e.g., cannabis users are different from us), and power asymmetry suggests that this group is “less than” the rest of society.^18^ Although the literature on stigma among individuals who use medicinal cannabis is still emerging, the literature on stigmatization among people with HIV and mental illness suggests stigma can pose a serious threat to engaging patients in care and achieving optimal outcomes.^19–21^ The intersection of stigma and the healthcare system may be relevant for CS who use cannabis, as cannabis stigma may negatively impact their interactions with healthcare. Although stigma appears salient among CS who use opioids, it is unclear if the same is true for cannabis.^6^

There is limited research on stigma among CS who use cannabis. One qualitative study with 24 CS who use cannabis found almost half the sample reported perception of stigma in the healthcare setting,^22^ while another study found fear of social stigma to emerge occasionally across CS.^23^ Although not specific to cancer, other qualitative studies on people who use cannabis for medicinal reasons suggest they hold self-perceptions as “junkies” or “potheads” and may not disclose use with doctors due to feared judgement.^24,25^ Indeed, among individuals who use medicinal cannabis, greater endorsement of anticipated stigma may be related to nondisclosure to providers.^26^ As non-judgmental and compassionate patient-provider communication is the cornerstone of effective healthcare, this is concerning.^27^ However, more research on cannabis stigma in the oncology population is needed.

The prior qualitative study on cannabis-related stigma among CS utilized semi-structured interviews and directly asked CS whether they had experienced stigma.^22^ While this approach yielded valuable insights, a broader investigation is warranted. The present study builds on this work by including both cannabis users and non-users, assessing indicators of stigma without explicitly prompting for it, and incorporating a quantitative measure of cannabis-related attitudes. More specifically, this study examines stigma concerning the patient-provider relationship, decisions to use or not use cannabis, and comparisons to opioid use.

## Methods

### Research Design

This study employed a convergent, parallel mixed methods design utilizing focus group and questionnaire data. Focus groups were separated such that CS who self-reported use of non-synthetic cannabis (i.e., excluding Dronabinol, etc.) at least once a week were grouped, and CS who do not use were grouped. Groups were facilitated by a clinical psychology doctoral student (SL).

### Participants and Recruitment

Participants enrolled in a large, multi-site study on cannabis in in cancer-related pain outcomes (see NCT06037681) were invited to participate if they expressed interest in other research opportunities. Participants were recruited from the Western New York and Eastern Pennsylvania regions. Eligibility for the parent study included age of 21 and older, self-identified African American/Black or White, diagnosed with a non-skin solid malignancy, received treatment within the past 3 years, and experienced cancer- or cancer-treatment related pain for >30 days. Additional inclusion criteria specific to this study were willingness/capability to participate in a recorded focus group. All participants provided written consent prior to participating and compensated $10 for completing the survey and $30 for the focus group. The Institutional Review Board at the State University of New York at Buffalo approved this study.

#### Quantitative methods.

Quantitative data specific to the present study were collected through REDCap and administered directly before the focus group.^28^

##### Demographics and Cannabis Use.

Demographic, cancer-related information, and cannabis use data from the parent study were extracted and linked to reduce participant burden. Cannabis use (collected monthly as part of the parent grant) from the last completed visit was used here.

##### Medical Cannabis Attitudes Scale.

The Recreational and Medical Cannabis Attitudes Scale (RMCAS) is a 12-item scale that assesses social beliefs, past beliefs, perceived risk, and legal restraints about cannabis use.^29^ The scale has two six-item subscales: attitudes towards medical and attitudes towards recreational cannabis use. Scores range from 6 to 30, with higher scores indicating more favorable attitudes. The two subscales have demonstrated adequate internal consistency with Cronbach’s alphas of .86 for the medical cannabis subscale and .91 for the recreational.^29^

#### Qualitative methods.

All focus groups were conducted virtually via a secure Zoom platform. Video recordings were stored on a secure, web-based server, and transcripts were obtained through Zoom’s transcription service. Inaccuracies were corrected based on the recordings. Identifiable information was deleted from the transcripts before analysis. All names below are pseudonyms.

The focus groups explored topics related to the decision to try cannabis, thoughts or concerns about use, conversations with providers, and how well they feel their pain symptoms are managed. Questions on the guide were informed by prior literature and the four components of stigma. The guide for CS who use cannabis was more explicit in asking about their thoughts, experiences, and perspectives, while groups with CS who do not use cannabis were more general about their perspectives on cannabis use. The word stigma was omitted from guides to minimize biased responses (i.e., making potentially stigmatized identities more salient can prime individuals to respond in a conforming way).^30^ AllCS were also asked about their experiences with and perspectives on opioids.

### Data Analyses

Quantitative analyses were conducted using the SAS platform. Descriptive statistics were analyzed to supplement qualitative data by building a comprehensive understanding of how self-reported, quantitative attitudes towards cannabis may align or differ from verbal reporting. Group differences in attitudes were evaluated using Wilcoxon non-parametric tests.

Qualitative analyses were conducted using Dedoose. Transcripts were analyzed using direct content analysis, in which research questions were informed by prior research or theory.^31^ A stepped process was utilized. First, we developed codes based on the four components of stigma (i.e., labeling, stereotyping, power asymmetry, and linguistic separation). Next, we identified new codes as they emerged. Upon completion of an initial independent review by the first author (SL), a second coder (RLA) conducted an independent review of 30% of transcripts. Discrepancies were resolved through discussion and content review. Memos were maintained by both coders to facilitate transparency. Themes were reviewed with several participants to ensure that our interpretations accurately reflected the topics discussed during groups.

## Results

### Quantitative results.

#### Participant Characteristics.

Overall, 23 CS participated in the focus groups, which included three “use” groups (n=5; n=3; n=2), and three “nonuse” groups (n=4; n=6; n=3). Recruitment ended when saturation was met (i.e., no novel information arose in focus groups). On average, participants were 56 years old, represented a range of cancer types and stages, and most were from New York State (Table 1). Most participants who used cannabis reported using it at least once per day and reported a balanced distribution across routes (Table 1).

#### Attitudes Towards Cannabis.

Participants demonstrated favorable attitudes towards cannabis, as indicated by both subscales on the RMCAS (Table 1). No significant differences in attitudes emerged between groups in the medical domain (Z=0.22, *p*=.41) or the recreational domain (Z=0.87, *p*=.19) on the nonparametric Wilcoxon test.

#### Qualitative Results

Although initial coding was based on the four components of stigma, it quickly became evident that, concerning cannabis use, other factors outweighed the possibility of judgment or stigmatization. Indeed, only three of the four components were identified. There was no indication of linguistic separation or being marginalized because of cannabis use. Codes were then developed to capture the experiences that appeared to buffer against stigma. Seven themes, in addition to Stigma, were identified, which encompassed three domains: Self-Advocacy and the Cancer Experience, Navigating Cannabis Use or Nonuse, and Opioids in Cancer Care. Representative quotes are presented in Table 2.

#### Stigma.

Although stigmatizing labels (e.g., “pothead”, “addict”) were occasionally mentioned, they were rarely used to suggest the experience of stigmatization of patients who use cannabis (i.e., in conjunction with power asymmetry, linguistic separation, or stereotyping). However, one CS who used cannabis reflected on how perceived stigmatized labeling may have compromised the patient-provider relationship (see “Liz”). While another CS described anticipated stigma, as suggested by the presence of labeling and some degree of stereotyping (see “Katy”).

While these excerpts reflect the hypothesized experiences of stigma among CS who use cannabis, these accounts appeared to be the exception rather than the norm among this sample. Others described more subtle expressions of judgements related to cannabis use, such as feeling hesitant to disclose their use in larger group settings (see “Jean”). Surprisingly, non-disclosure of use to providers due to fear of judgment did not emerge across focus groups. Rather, some CS described not disclosing their use to providers for other reasons, seemingly unrelated to fear of being stigmatized. For example, one CS did not tell her provider about using cannabis because she “didn’t care” (see “Ruth”). Another expressed that she did not see a benefit in disclosing her use to her provider, citing that it would not change her decision to use (see “Laurie”). This may be an indirect way to prevent the experience of stigma, particularly if CS anticipate disapproval from providers. However, this cannot be determined or ruled out based on these quotes.

### Self-Advocacy and the Cancer Experience (See Table 2).

#### Identity, judgement, and inequities.

This theme captured how CS spoke about the unique challenges they face as a population that requires frequent engagement with providers. For example, several CS described an internal experience of wanting to be a “perfect patient”, which often led to minimizing symptoms to avoid burdening providers.

Additionally, some reflected on how their identities shaped their experiences as patients. Among those who identified as women, a common tension emerged between the desire to not “burden” their providers by disclosing pain while recognizing that open communication is necessary to receive effective pain management.Additionally, several patients expressed their perspective of being a Black or African American patient in the healthcare setting, particularly how it related to providers believing the extent of their pain.

Finally, the topic of judgement from other cancer survivors came up during one of the focus groups. One patient referenced how other CS would be more likely to judge you for eating peanut butter than using cannabis, suggesting that consumption of other things (e.g., processed foods) may evoke more judgment than cannabis (see “Denise”).

#### Cancer as a Unique Condition: Special Treatment and Exemptions.

Another general theme pertained to the experiences of having a cancer diagnosis. For example, it appeared to be a commonly shared sentiment that CS may be excused from potential judgment about choosing to use cannabis because of their illness.

#### Patient Empowerment and Self-Directed Pain Management.

This theme reflected both patients perceived need to, and empowerment in, navigating their conditions. The most salient code to emerge was patient empowerment. Many patients described learning how to advocate for themselves based on the perception that others do not care and/or do not have the time. Some spoke about the disconnect between a provider being the expert in medicine, and the patient being the expert in their body and that ultimately, they have to make decisions based on what was best for their body. Furthermore, another frequently coded topic related to patients rejecting judgment, expressing a deep belief that others do not understand what it is like to have cancer. As such, views and/or opinions of family, friends, and healthcare providers are often discounted.

### Opioids in Cancer Care (See Table 2)

#### Weighing Cannabis Against Opioid Risks and Concerns.

This theme reflected decisions to use cannabis in the context of concerns surrounding opioids. Some explained that their decision to use cannabis was related to their views of opioids, citing a disinterest in using opioids and a desire for alternatives.

#### Navigating Opioid Use: Pain, Stigma, and Fears of Addiction.

This theme, which emerged in all focus groups, captured the complex relationship that CS have with opioids. Notably, the word “stigma” was mentioned frequently in the context of opioids. Several CS indicated a struggle to balance effective pain relief with fears of dependence, often driven by personal accounts of addiction and/or societal views of opioids. Indeed, this conflict arose in real-time during a focus group in which two CS demonstrated more negative views of opioids, while one CS expressed a more positive perspective.

Several patients, particularly ones with advanced disease expressed acceptance of their need for opioids, and frustration and/or disappointment with the way that opioids are controlled. For example, one CS described the way he felt when his pills were counted, and being questioned by a medical provider.

### Navigating Cannabis Use or Nonuse (See Table 2)

#### When and Why Patients Choose to Use Cannabis.

This theme reflects how CS made decisions about when to use cannabis. This theme captured CS perspectives on cannabis’ effectiveness at relieving certain symptoms, and reasons why not to use. Some CS not currently using cannabis described either trying it recreationally before, and not liking the way it made them feel, or found that it did not help with symptoms. Others who use cannabis noted how cannabis has helped with sleep, pain, anxiety, and served as a “distraction;” one CS described, “*it’s a relaxing way to alter my viewpoint on some of the things that are going on in life that aren’t so happy.”*

#### Navigating Medicinal Cannabis Use: Concerns and Considerations.

This theme captured the internal and external concerns CS expressed about cannabis use. For example, when asked if anyone had questioned their cannabis use, “Dorothy” explained that she has become dependent on cannabis. When describing “dependent,” she elaborated that, “I*f I’m running low, and I don’t have any, I panic*”. Another CS reflected on how others may perceive her because she is no longer “sick,” but is still using cannabis for other reasons (e.g., anxiety, PTSD symptoms), suggesting potential anticipated stigma. Other discussions included dosing considerations, trusting product labels, and tolerance. As one CS described, “…*the few times that I have backed off is because I would see that my tolerance was going up, and that I needed to take a break because it wasn’t having the same effect*.” Another CS described, “*I have to use more than I used to for the appropriate results*.”

### Integrating Qualitative and Quantitative Results.

To evaluate how attitudes towards cannabis mapped onto qualitative data, we selected 3 participants each from the lower and upper quartiles on RMCAS (i.e., scores <17 and scores >24, respectively) and evaluated relevant quotes (Table 3). Even CS who reported less positive attitudes towards cannabis tended to express an accepting or neutral view of others using cannabis. Consistent with the Patient Empowerment and Self-Directed Pain Management themes, CS expressed that people should be free to make their own medical decisions. CS who reported more positive attitudes expressed stronger beliefs about the importance of having access to cannabis, including a degree of advocacy about why others should be more open-minded about cannabis use.

## Discussion

The present study assessed the presence of cannabis-related stigma among CS who use or may consider using cannabis to manage symptoms. Given the complicated history of cannabis, assess implications on the patient experience is important. Our study suggests that the experience of stigma may exist for some CS, but for many, other experiences seem to eliminate or overshadow the perception and/or experience of cannabis-related stigma. Notably, we found that opioid stigma and negative views of opioid use persist, and these views may influence decisions to use cannabis.

Coding for stigma through the theoretical lens of the four-component model (i.e., labeling, separation, stereotyping, power asymmetry) revealed few reports of experienced stigma. As several CS described their decision not to disclose cannabis use to their provider, this may reflect a subcategory of stigma referred to as anticipated stigma.^32^ Anticipated stigma refers to an individual’s belief that they will experience discrimination or judgement in the future, which may prevent CS from disclosing cannabis use, as seen in other stigmatized populations.^33^ This is concerning given herb-drug interactions and missed opportunities to recommend safer methods of consumption (e.g., edibles instead of smoked flower).^34^

Prior literature highlights factors like a lack of physicians’ inquiry and patients’ perception that disclosure is irrelevant leading to nondisclosure of complementary alternative medications (CAM).^35^ However, CAM was not specific to cannabis use, and more research is needed on barriers and facilitators of open patient-provider discussions concerning cannabis. Given current and prior CAM disclosure findings, it is recommended that providers be clear about their intentions for inquiring. For example, providers may explain that they are not asking to discipline or refute the fact that cannabis helps; rather, open communication about all substances facilitates the best possible care. Further, recent guidelines recommend that clinicians routinely and nonjudgmentally ask patients about cannabis use, or consideration of use, and provide CS with unbiased, evidence-based educational resources.^36^ Additionally, findings related to self-directed pain management and empowerment support the notion that CS want to be involved in treatment decision-making.^37^

It was evident throughout groups that CS related to one another and developed strong camaraderie. This may partly explain why CS who do not have positive views of cannabis still feel accepting or neutral about other CS who use cannabis. Indeed, the mutual experience of having cancer may facilitate an in-group identification and a sense of nonjudgmental acceptance compared with friends/family.^38^ This within-group identification may protect CS against stigma, as sense of belonging has been shown to buffer the internalization and possible effects of stigma.^39^ Additionally, CS described a figurative “cancer shield” that seemed to protect them against stereotyping or judgements that others may receive for using cannabis. Nevertheless, pervasive opioid stigma appeared to penetrate this figurative shield and factored into the symptom management decision-making process.

Unfortunately, this finding corroborates other literature that highlights opioid stigma as a significant barrier to cancer-pain management.^40–42^ Combined with CS’s doubts about providers’ ability to empathize, this suggests that non-judgmental, compassionate patient-provider communication is essential. Many CS do not feel heard and are misunderstood by healthcare providers, which is reflected in our finding that patients feel like they must “take matters into their own hands,” especially surrounding their symptom management.^7,43^

These findings differ somewhat from a previous qualitative study in which CS frequently reported stigma.^22^ However, a quarter of their sample from the Eastern U.S. denied any stigma, which was consistent with our Eastern U.S. sample, suggesting regional differences in cannabis-related stigma. In contrast, a 2024 qualitative study on medicinal cannabis use among CS in Australia also found positive attitudes towards cannabis, especially with attention to the perceived necessity of use among CS,^44^ which is similar to our sample. Rather than asking *if* CS who use cannabis experience stigma, it may be more appropriate to ask *which* CS experience stigma and in what context. Although outside the scope of this paper, prior research identified correlates like gender, depression, and self-esteem that are associated with stigma about one’s cancer diagnosis.^45^

This study has several limitations. First, participants who enroll in cannabis-related studies may inherently be more comfortable discussing the topic. Second, the sample reflects perspectives from CS in two states that have medical cannabis laws and may not reflect perspectives from other areas of the United States or in the world. Third, focus groups (versus individual interviews) may raise the risk of social desirability bias and group conformity effects, which may have limited CS from sharing their individual, authentic views. Finally, our sample size was small. While we used non-parametric tests, the possibility of a type II error exists.

In sum, this study suggests that while cannabis stigma may not be commonplace for CS, some do experience it. Additionally, anticipated stigma may contribute to nondisclosure of use. This, combined with broader issues related to CS perception of needing to manage their own symptom suggests that additional research on how healthcare providers can facilitate effective and empathetic conversations about cannabis use is needed. Further, opioid stigma is widespread and impacts medical decision-making processes. Understanding the factors that make CS susceptible to stigma will be critical to prevent the downstream effects. Furthermore, the development of a standardized medicinal cannabis assessment tool is warranted to support research on these factors.

## Figures and Tables

**Table 1.** Descriptive Results

	Cannabis Use Status		

Characteristic	Yes (*n*=10)	No (*n*=13*)*	Full sample (*n*=23)

Age *M* (SD)	57.1 (8.8)	55.2 (11.7)	56.0 (10.4)

Cancer Type			
*Breast*	5	7	12
*Gastrointestinal*	1	2	3
*Genitourinary*	1	1	2
*Gynecological*	1	2	3
*Myeloma*	1	0	1
*Thoracic*	1	1	2

Cancer Stage			
*1*	1	5	6
*2*	1	3	4
*3*	3	2	5
*4*	4	3	7

Primary Type of Cannabis			
*Edibles* ^a^	4		
*Flower (smoked)*	3		
*Oil (ingested)* ^b^	3		

Average Frequency of Use			
*5 – 6 Days a Week*	1		
*Once a Day*	6		
*More than Once a Day*	3		

Attitudes Toward Medical Cannabis^c^	22.0 (22.0, 25.0)	23.0 (16.0, 24.0)	23.0 (20.0, 24.0)

Attitudes Towards Recreational Cannabis^c^	22.5 (21.0. 24.0)	22.0 (14.0, 24.0)	22.0 (15.0, 24.0)

MCLaSS^d^			
*New York (n=18)*			78
*Pennsylvania (n=5)*			52

a Patients reported a mix of gummies, candy, and baked goods

b Two patients reported use of tinctures, and one reported use of Rick Simpson Oil

c Scores are presented as median (IQR)

d Medicalization of Cannabis Laws Standardized Scale; Scores are presented as summary scores

**Table 2.** Qualitative Results and Exemplary Quotes

Theme ^a^	Number of Excerpts	Example Quotes
Cannabis Stigma	10	*“When they gave me push back [about asking for cannabis], it was like I was doing something wrong, or I was some kind of drug addict, and I didn’t really need it...” “I felt like I was asking for something that I shouldn’t be asking for. That it was some outrageous request. I didn’t like the fact that she didn’t want to help me, and that she referred me to somebody else who wasn’t even my actual doctor. And ever since then, I’ve had a lot of issues with her, just talking to her about things and sharing things, and I just feel like she’s very judgmental.”* • “Liz,” uses cannabis, stage 2 breast cancer *“My parents go to the same primary doctor. I think, too, not that they care but I just felt like I didn’t want to be judged, and had put in my chart that you know, I’m a druggie, or whatever.* • “Katy,” uses cannabis, stage 3 gynecologic cancer *“...so, it’s very acceptable, and yet at the same time, would I want to tell people at a work party necessarily that I’m doing it? I don’t know. I think twice about that. The judgement’s there.”* • “Jean,” uses cannabis, multiple myeloma *“I can speak to the fact that I’ve never discussed it with my oncologist. Part of it was because I didn’t want him to tell me it wasn’t a good idea.... I don’t necessarily need advice from somebody who’s not going through what I’m going through and living in my body.”* • “Laurie,” uses cannabis, stage 4 genitourinary cancer
Self-Advocacy and the Cancer Experience		
Identity, Judgement, and Inequities	20	*“I feel like I have this tendency to like need to be like the perfect patient in some way or like that I need to be like perfect and easy. That’s my fault.”* *“We shut up, and we do the thing, and we act like the perfect patient, and women in general, I think, are pleasers, you know? Like we want to make everybody happy, and we want to be good at everything and make sure other people’s needs are taken care of and forget about the fact that we’re the sick people..”* • “Jane,” does not use cannabis, stage 3 gynecological cancer *“...just my experience as a black man, but also going through the cancer journey is you have to tell them you’re in pain... I don’t want to get to the point where it’s pain that I can’t manage, or they’re thinking that I’m not in pain, and I don’t want them to believe that I’m not in pain.”* • “Marcus,” does not use cannabis, stage 2 gastrointestinal cancer *“It is funny like they’ll talk bad about you if you eat peanut butter, but not if you use cannabis.”* • “Denise,” does not use cannabis, stage 1 gynecologic cancer
Cancer as a Unique Condition: Special Treatment and Exemptions	28	*“Every once in a while I get a little feedback from somebody that feels like they are judging. But you know what, they don’t judge because I have the cancer where before they would have. I think now that I have cancer, I tell everybody that I am on prescription marijuana, and everybody’s fine with it, but most people laugh about it.”* • “May,” uses cannabis, stage 3 breast cancer *“I don’t think you get [weird] reactions. Maybe if you’re using pot, or just to get high or opiates just to be high, then maybe you get that reaction. But when people see how crippled you are and how much you’re wincing in pain, and they know you have cancer, I don’t see it.”* • “Jean,” uses cannabis, multiple myeloma
Patient Empowerment and Self-Directed Pain Management	75	*“You know, I guess this is my second cancer, and so I am of the opinion that whatever I need to do to save my life, to feel better, I’m going to do it. I don’t care.”* • “Gloria,” does not use cannabis, stage 1 breast cancer *“You gotta take care of yourself. You have to be your own advocate, and only you can determine what that is... There should still be some joy and pleasure in life, and if that’s [cannabis] what gives it to you, then who is a doctor to tell you that you can’t do it.”* • “Laurie,” uses cannabis, stage 4 genitourinary cancer *“Right now, I’m starting to be more of an advocate for myself because I see everyone else not caring.”* • “Julie,” uses cannabis, stage 4 breast cancer *“I did worry about that, having my name in their system, and like who’s gonna find out about it [cannabis use], and you know, Big Brother and all that kind of crap. But now it’s like, ‘you know what, it’s legal, and if you don’t like that that I do it, screw you’.”* • “Katy,” uses cannabis, stage 3 gynecologic cancer
Opioids in Cancer Care		
Weighing Cannabis against Opioid Risks and Concerns	34	*“What is this [opioids] even good for? Like I don’t even understand, like it’s not taking any of my pain away. It’s just making me feel funny. So then, that was my decision that I was going to start doing marijuana.”* • “Julie,” uses cannabis, stage 4 breast cancer *“I do think that’s why I asked for medical marijuana, because I was trying not to take opiates.”* • “May,” uses cannabis, stage 3 breast cancer *“Opioids are just crazy to me that they still prescribe them, and then they’re like, ‘oh, well, how come there’s so many people addicted?’ well, you know, it’s what you’re giving them. So, I think it’s [cannabis] a better alternative, in my opinion.”* • “Katy,” uses cannabis, stage 3 gynecologic cancer *“...my goal is to fully be off the opioids, like taper down whatever I need to do but to get off the opioids. I don’t feel like I would ever be ashamed to say like, Oh yeah, I eat a gummy once in a while, or, you know, smoke pot or whatever.’ There’s just not that stigma, or shame behind it.”* • “Jane,” uses cannabis, stage 3 gynecological cancer
Navigating Opioid Use: Pain, Stigma, and Fears of Addition	75	*“...and she was treating me like I’m some kind of drug addict, and I’m overusing my pills, and I need to take the pills the way they’re prescribed. I got so mad at her, I was swearing at her because it’s like, no, you don’t get it. You guys are making me cut these pills in half. They’re crumbing, and I got to take what I can take.”* • “Ted,” does not use cannabis, stage 4 gastrointestinal cancer *“...people just get addicted. And you hear so much like, ‘Oh, he had back surgery, and all of a sudden he was addicted to painkillers,’ or whatever, you know? And it’s just like no one sets out trying to become an addict. So at what point am I an addict? Like I don’t know, you know? Like ‘Oh, shit! I’m addicted!’”* • “Jane,” does not use cannabis, stage 3 gynecological cancer *“You just kind of worry about people in society judging you that you’re getting these things.”* • “Marcus,” does not use cannabis, stage 2 gastrointestinal cancer *“I think there’s more of a stigma on that nowadays. People look at you a lot differently when you use those painkillers and are more of a stigma all of a sudden.”* • “May,” uses cannabis, stage 3 breast cancer *“And I agree with “May” that there’s definitely a stigma about them. Now you’re kind of looked at like, maybe you’re a drug addict if you take too many of them.”* • “Liz,” uses cannabis, stage 2 breast cancer
		Navigating Cannabis Use or Nonuse
When and Why Patients Choose Cannabis	53	*“I don’t like to not be in control of everything about myself. I’ve tried it in the past recreationally when I was much younger, but I really didn’t have fun like everybody else did... So for just me personally, it’s not a choice for me.”* • “Angela,” does not use cannabis, stage 1 breast cancer *“I just use it for sleep, and I was using it for pain management in the beginning, but the pain has gone away at this point with the chemo treatments, so I just use it for sleep”* • “Bruce,” uses cannabis, stage 4 gastrointestinal cancer *“It’s a distraction. It definitely takes your mind away from your worries a lot of the time for me. It makes me feel better. It definitely helps with some of the pain that I have in my feet right now.”* • “Laurie,” uses cannabis, stage 4 genitourinary cancer *“Well I think it’s fine, if that’s what helps you. For me, I don’t do cannabis because it doesn’t help me at all.”* • “Ted,” does not use cannabis, stage 4 gastrointestinal cancer • •
Navigating Medicinal Cannabis Use: Concerns and Considerations	140	*“... and you know, just talking to y’all, I can honestly say and I never given it this type of thought that I have become dependent on my marijuana, and when I don’t have it, I’m irritated...I might have to go half a day or something, but in that time that I don’t have it, it’s like something’s missing.”* • “Dorothy,” uses cannabis, stage 1 breast cancer *“I’m questioning my usage of it, because I’m taking too much sometimes, and not enough other because I don’t know.. I think it varies, and I’m not sure if I’m confident in what you know, the people selling it are saying... I don’t believe them. The only way I find out it by trying it.”* • “Ruth,” uses cannabis, stage 3 breast cancer *“I’m still using cannabis for other reasons, and I don’t think that it’s as accepted [as using for cancer]. It’s still, people are kind of wondering, ‘why are you smoking? Why are you smoking so much? Why are you still using it if you’re not sick? That kind of thing. And I do feel like I have to hide it sometimes, and I don’t like feeling that way.”* • “Liz,” uses cannabis, stage 2 breast cancer

a Most themes emerged in all six focus groups, except for *Identity, Judgement, and Inequities* which only emerged in four, and *Cancer as a Unique Condition: Special Treatment and Exemptions* which emerged in five groups.

**Table 3.** Qualitative and Quantitative Attitudes on Cannabis

Participant	Recreational and Medical Cannabis Attitudes Scale Score	Key Quote	Qualitative Theme
**Lower Quartile RMCAS**			
“Gloria,” does not use, stage 1 breast	8.5	*“I grew up extremely poor, and so I have not seen any positive effects from any type of drug... I get alarmed because, you know, I see children getting out of cars, and when they open the door they’re driving and the whole car is filled with smoke...However, I’m a person who believes in your right to do what you need to do, so I’m not going to judge anyone else for doing whatever they need to do.”*	When and Why Patients Choose to Use Cannabis
“Denise,” does not use, stage 1 gynecologic	13.0	*“... I agree, I don’t know why we would deny somebody medication [cannabis] that helps them... and when all of your evidence is based on what you learned in the bathroom in 1978 in high school is just kind of ignorant.”*	When and Why Patients Choose to Use Cannabis
“Sue,” does not use, stage 3 breast	15.0	*“I don’t know how I feel about it [cannabis]... I tried it in my younger years, and I really didn’t like the way it made me feel.”*	Navigating Medicinal Cannabis Use: Concerns and Considerations
**Upper Quartile RMCAS**			
“Julie,” uses cannabis, stage 4 breast	24.5	*“I’m glad that it [cannabis] has become legal, and just, people need to be more open of it. You know, I can see the effects, what it does to young kids, which I don’t like, but I can see the effects, how it affects me, and my body.”*	Navigating Medicinal Cannabis Use: Concerns and Considerations
“Laurie,” uses cannabis, stage 4 genitourinary	24.5	*“It [cannabis] makes me feel better. It definitely helps with some of the pain that I have in my feet right now. And I just find that it’s a relaxing way to alter my viewpoint on some of the things that are going on in life that aren’t so happy...and I don’t really care if anybody else thinks it’s good, bad, or indifferent. It’s none of their business, that’s kind of my bottom line.”*	Patient Empowerment and Self-Directed Pain Management
“Ted,” does not use, stage 4 gastrointestinal	25	*“Well, I think it’s [cannabis] fine, if that’s what helps you. For me, I don’t do cannabis because it doesn’t help me at all. And it’s because of the silly views of society that say ‘drugs are bad’ but they don’t really investigate anymore. They just take what they’ve been taught about cannabis and go ‘this must be’.”*	Navigating Medicinal Cannabis Use: Concerns and Considerations
